# Effect of thyroid hormone concentration on the transcriptional response underlying induced metamorphosis in the Mexican axolotl (*Ambystoma*)

**DOI:** 10.1186/1471-2164-9-78

**Published:** 2008-02-11

**Authors:** Robert B Page, Stephen R Voss, Amy K Samuels, Jeramiah J Smith, Srikrishna Putta, Christopher K Beachy

**Affiliations:** 1 Department of Biology, University of Kentucky, Lexington Kentucky, 40506 USA; 2 Spinal Cord and Brain Injury Research Center, University of Kentucky, 40506 USA; 3 Department of Biology, Minot State University, Minot, North Dakota, 58707 USA; 4 Amphibian Growth Project, Minot State University, Minot, North Dakota, 58707 USA

## Abstract

**Background:**

Thyroid hormones (TH) induce gene expression programs that orchestrate amphibian metamorphosis. In contrast to anurans, many salamanders do not undergo metamorphosis in nature. However, they can be induced to undergo metamorphosis via exposure to thyroxine (T_4_). We induced metamorphosis in juvenile Mexican axolotls (*Ambystoma mexicanum*) using 5 and 50 nM T_4_, collected epidermal tissue from the head at four time points (Days 0, 2, 12, 28), and used microarray analysis to quantify mRNA abundances.

**Results:**

Individuals reared in the higher T_4 _concentration initiated morphological and transcriptional changes earlier and completed metamorphosis by Day 28. In contrast, initiation of metamorphosis was delayed in the lower T_4 _concentration and none of the individuals completed metamorphosis by Day 28. We identified 402 genes that were statistically differentially expressed by ≥ two-fold between T_4 _treatments at one or more non-Day 0 sampling times. To complement this analysis, we used linear and quadratic regression to identify 542 and 709 genes that were differentially expressed by ≥ two-fold in the 5 and 50 nM T_4 _treatments, respectively.

**Conclusion:**

We found that T_4 _concentration affected the timing of gene expression and the shape of temporal gene expression profiles. However, essentially all of the identified genes were similarly affected by 5 and 50 nM T_4_. We discuss genes and biological processes that appear to be common to salamander and anuran metamorphosis, and also highlight clear transcriptional differences. Our results show that gene expression in axolotls is diverse and precise, and that axolotls provide new insights about amphibian metamorphosis.

## Background

Amphibian metamorphosis is generally characterized by dramatic and conspicuous developmental changes that are necessary for larvae to function as terrestrial adults. The morphological, behavioral, and physiological changes that occur during metamorphosis are associated with increases in thyroid hormone (triiodothyronine, T_3 _and thyroxine, T_4_; TH) [1,2] and RNA synthesis [3]. These events are interconnected; at metamorphosis, tissue-specific concentrations of TH activate and repress transcriptional networks within target cells that in turn regulate new patterns of development [4]. Many genes that are associated with molecular and morphological events during metamorphosis have been identified from studies of anurans, and in particular *Xenopus laevis*. In contrast, little is known about patterns of gene expression during salamander metamorphosis.

Although anuran and salamander metamorphosis are regulated by many of the same endocrine factors, there is considerable developmental variation between these groups. Most conspicuously, some salamanders do not undergo a complete metamorphosis in nature. These salamanders are called paedomorphs because they retain larval characteristics into the adult stage, and because genetic and phylogenetic evidence suggests that they evolve from metamorphic ancestors [5,6]. Paedomorphosis in the Mexican axolotl (*Ambystoma mexicanum*) is associated with low hypothalamic-pituitary-thyroid (HPT) activity and differential sensitivity of tissues to TH that results in some cryptic biochemical and molecular changes, but not the complete suite of morphological changes seen in related, metamorphic tiger salamanders. Interestingly, *A. mexicanum *can be induced to undergo anatomical metamorphosis by administering TH and endocrine factors that function upstream of TH synthesis [7,8]. The axolotl provides an excellent alternative to anuran systems because metamorphosis can be precisely induced and studied in juveniles or adults that are not developing toward a metamorphic outcome.

Functional genomic approaches are beginning to reshape the way transcription is conceptualized during amphibian metamorphosis [9-11]. The transcriptional program for tissue regression, remodeling, and organogenesis is significantly more complicated than was initially predicted for *Xenopus *[12-15]. Previously, we used microarray technology to identify keratin biomarkers for T_4 _induced metamorphosis in the integument (epidermis) of the Mexican axolotl [11]. We showed that 50 nM T_4 _induces a complex transcriptional program and axolotls complete metamorphosis with no mortality. Interestingly, this T_4 _concentration is known to affect gene expression and mortality in anurans [16-18] and it is higher than T_4 _concentrations estimated in the serum of spontaneously metamorphosing salamanders. For example, Larras-Regard et al. [2] reported 28 nM as the maximum serum T_4 _level in *Ambystoma tigrinum*, a close relative of the axolotl that typically undergoes metamorphosis. To further investigate the effect of T_4 _concentration on induced metamorphosis in the Mexican axolotl, we report the results of a second microarray experiment that induced metamorphosis using a much lower concentration of T_4 _(5 nM). Using 5 and 50 nM T_4 _microarray datasets, we describe the temporal transcriptional response of T_4 _induced metamorphosis and specifically address the following question: Does T_4 _concentration affect morphological metamorphosis and gene expression in the axolotl? We discuss the biological significance of some of the differentially expressed genes (DEGs) that were identified and the relationship between salamander and anuran metamorphic gene expression programs.

## Results

### Effect of T_4 _concentration on morphological metamorphosis

During T_4 _induced metamorphosis, Mexican axolotls progress through developmental stages (0–IV) [19] that are defined by the resorption of the upper and lower tailfins, dorsal ridge, and gills. We staged all axolotls after 0, 2, 12, and 28 days of T_4 _treatment. No metamorphic changes were observed after two days of T_4 _treatment and thus axolotls from both T_4 _treatments were assigned to Stage 0. At Day 12, morphological changes were observed in 50 nM T_4 _treated axolotls (Stages I and II) but 5 nM T_4 _treated axolotls were indistinguishable from control animals (Stage 0). At Day 28, axolotls reared in 50 nM T_4 _had fully resorbed tailfins and gills, and thus had completed morphological metamorphosis (Stage IV). Between Days 12 and 28, 5 nM T_4 _treated axolotls initiated metamorphosis but did not complete all morphological changes by Day 28 (Stage III). On average, individuals complete metamorphosis after 35 days in 5 nM T_4 _(unpublished data). Thus, a low concentration of T_4 _delays the initiation timing of morphological metamorphosis but not the length of the metamorphic period.

### Gene expression in the absence of T_4_

Our first set of statistical analyses tested control axolotls for temporal changes in mRNA abundance that were independent of T_4 _treatment [see Additional files 1, 2]. Temporal changes are expected if patterns of transcription (gene expression) change significantly over time as salamanders mature, or if there are uncontrolled sources of experimental variation. After adjusting the false discovery rate (FDR) to 0.05, none of the probe-sets (genes) on the custom *Ambystoma *GeneChip were identified as significantly differentially abundant as a function of time. Thus, we found no statistical support for differential gene expression among control animals.

### Gene expression in the presence of T_4_: 5 versus 50 nM

**Figure 1:**
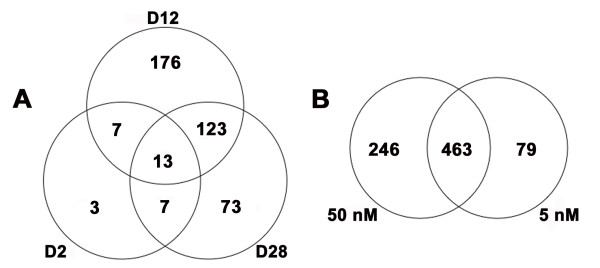
**Results of the statistical analyses conducted on the microarray data**. (A) Venn diagram showing the DEGs identified at specific time points by contrasting the two T_4 _concentrations and imposing fold change criteria. D2 = Day 2, D12 = Day 12, and D28 = Day 28. (B) Venn diagram depicting the relationship between DEGs that were identified via the 5 and 50 nM regression analyses and imposing fold change criteria. For panel B, fold change values are relative to Day 0.

At each of the times that we estimated mRNA abundances during T_4 _induced metamorphosis, the number and diversity of genes that were differentially expressed between the 5 and 50 nM T_4 _treatments differed (Figure 1A) [see Additional files 3, 4]. A total of 402 DEGs that differed by ≥ two-fold at one or more of our sampling times were identified among all day by T_4 _treatment contrasts (Figure 1A) [see Additional files 3, 4]. We identified 30 DEGs as early as Day 2 (Table 1), and eighty percent of these DEGs were up regulated in 50 nM T_4 _relative to 5 nM T_4_. This small group of early response genes was statistically associated with the amino acid transport and amine transport ontology terms. Additional gene functions of these early responding genes include epithelial differentiation, ion transport, RNA processing, signal transduction, and apoptosis/growth arrest. We note that no differentially expressed transcription factors were identified as DEGs at Day 2, and neither thyroid hormone receptor (TR; alpha and beta) was identified as differentially expressed at any time point in our study. At Day 12, when axolotls in 50 nM T_4 _were undergoing dramatic tissue resorption events and axolotls in 5 nM T_4 _were indistinguishable from controls, we identified the greatest number of DEGs between the T_4 _treatments (*n *= 319; Figure 1A). An approximately equivalent number of up and down regulated DEGs were identified [see Additional files 3, 4]. These genes were enriched for functions associated with epidermis development, carbohydrate metabolism, ectoderm development, response to chemical stimulus, negative regulation of cell proliferation, response to abiotic stimulus, negative regulation of biological process, development, and organ development. By Day 28, when axolotls in 50 nM T_4 _had completed metamorphosis and axolotls in 5 nM T_4 _were continuing to show morphological restructuring, we identified 216 DEGs that differed between the T_4 _treatments (Figure 1A). Of the identified DEGs, 76% were down regulated in 50 nM T_4 _relative to 5 nM T_4 _[see Additional files 3, 4]. DEGs identified at Day 28 were associated with response to pest, pathogen, or parasite, negative regulation of cellular process, positive regulation of physiological process, response to stress, response to stimulus, transition metal ion transport, di-, tri-valent inorganic cation transport, response to other organism, immune response, development, organismal physiological process, muscle contraction, response to biotic stimulus, and response to bacteria. The functional categories that we identified show that the axolotl epidermal transcriptional response to T_4 _is complex, involving hundreds of DEGs.

**Table 1:** Genes identified as differentially abundant between 5 and 50 nM T_4 _at Day 2

Gene	Probe-set ID	Contig ID	*t*	*P*	Fold Change
*Actin, alpha, cardiac muscle*	SRV_12180_x_at	TC01592	-4.17	0.0004	-8.91
N/A	SRV_07408_at	MC07291	-2.49	0.0209	-2.83
*Solute carrier family 7, member 3*	SRV_04996_at	MC04879	-2.33	0.0295	-2.47
*Lipopolysaccharide-induced TNF factor*	SRV_05921_at	MC05804	-2.66	0.0144	-2.38
N/A	SRV_09913_at	MC09796	-2.98	0.0069	-2.22
*Regulator of G-protein signaling 5*	SRV_01913_a_at	MC01801	-2.35	0.0283	-2.18
*Cadherin 1, type 1, E-cadherin*	SRV_11961_at	TC01382	4.19	0.0004	5.65
ATP-binding cassette, sub-family B, member 4	SRV_10722_at	TC00206	3.11	0.0051	5.64
N/A	SRV_05709_at	MC05592	3.84	0.0009	4.79
N/A	SRV_05710_a_at	MC05593	4.93	< 0.0001	4.78
N/A	SRV_10033_at	MC09916	3.90	0.0008	3.25
*Solute carrier family 6, member 14*	SRV_03196_a_at	MC03084	2.79	0.0108	3.00
N/A	SRV_10338_at	MC10221	2.69	0.0134	2.63
N/A	SRV_07637_at	MC07520	2.80	0.0104	2.44
*Transglutaminase 1*	SRV_00309_at	MC00197	2.69	0.0134	2.28
*Phosphoenolpyruvate carboxykinase 1*	SRV_01492_at	MC01380	2.57	0.0177	2.24
N/A	SRV_08888_at	MC08771	2.28	0.0324	2.04
N/A	SRV_07731_at	MC07614	2.39	0.0258	2.04
*Adipose differentiation-related protein*	SRV_00744_a_at	MC00632	2.06	0.0501	1.89
*Chemokine ligand 5*	SRV_01687_at	MC01575	2.81	0.0102	1.72
N/A	SRV_07816_a_at	MC07699	2.32	0.0304	1.68
N/A	SRV_05752_at	MC05635	3.11	0.0051	1.64
*Heterogeneous nuclear ribonucleoprotein L-like*	SRV_05212_a_at	MC05095	2.59	0.0168	1.57
*Transmembrane protein 79*	SRV_04923_at	MC04806	2.19	0.0396	1.53
N/A	SRV_07996_at	MC07879	2.81	0.0102	1.51
N/A	SRV_06589_at	MC06472	2.05	0.0525	1.44
N/A	SRV_05658_a_at	MC05541	2.17	0.0410	1.43
*Solute carrier family 11, member 1*	SRV_00441_at	MC00329	2.32	0.0301	1.34
*Dystroglycan 1*	SRV_02177_at	MC02065	2.44	0.0232	1.30
*Mitogen-activated protein kinase kinase 3*	SRV_05297_at	MC05180	2.32	0.0303	1.20

N/A refers to genes that do not have established orthologies to human. Probe-set ID represents the unique identifier for each probe-set on the custom *Ambystoma *GeneChip. Contig ID represents the Sal-Site [52] identifier for contigs associated with each probe-set. All fold changes are relative to the 5 nM dataset. Thus positive values are up regulated in 50 nM samples relative to 5 nM samples and negative values are down regulated in 50 nM samples relative to 5 nM samples.

**Figure 2:**
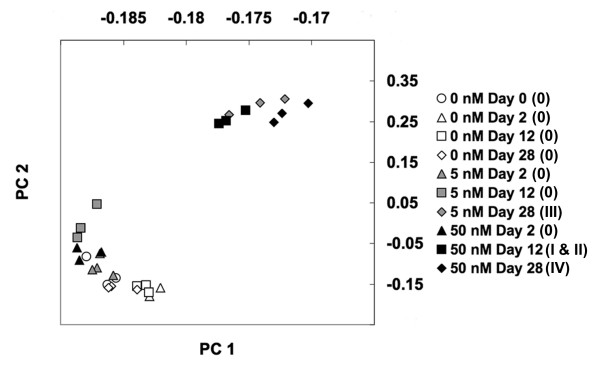
**Results of the principal component analysis**. Scatter plots of the 30 GeneChips based on the rotations of their first two principal components (PC 1 and PC 2). PCA was performed on all 3688 probe-sets that were available for significance testing. PC 1 and PC 2 account for 89.2% and 6.2% of the variance respectively. Cano-Martinez stages are listed in parenthesis in the legend.

To further explore the effect of T_4 _on gene expression and morphological metamorphosis we conducted a principal component analysis (PCA). This analysis shows that global gene expression and morphological metamorphosis are strongly correlated, but there is little or no correlation between gene expression and T_4 _treatment (Figure 2). This suggests that after metamorphosis was initiated within T_4 _treatments, molecular and morphological events were coordinately regulated. T_4 _concentration affected the onset timing of metamorphosis in axolotls, but not the sequence of transcriptional and morphological events that define this process.

### Modeling the transcriptional response of genes during induced metamorphosis

**Figure 3:**
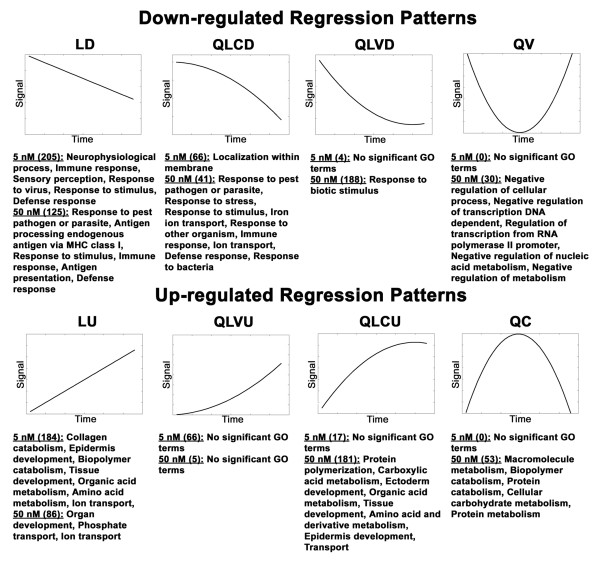
**Generalized regression patterns**. The generalized regression patterns recovered by the methodology described in Liu et al. [52]. The units and values of the axes are arbitrary. The number of probe-sets in each category is listed in parentheses by concentration. Significantly enriched biological process gene ontology terms are listed by concentration and pattern. In some cases specific terms were too abundant and were thus summarized via a broader term. Abbreviations are as follows LD = linear down, QLCD = quadratic linear concave down, QLVD = quadratic linear convex down, LU = linear up, QLCU = quadratic linear concave up, QLVU = quadratic linear convex up, QC = quadratic concave, and QV = quadratic convex. A ninth pattern (Flat) that describes null results is not shown.

**Figure 4:**
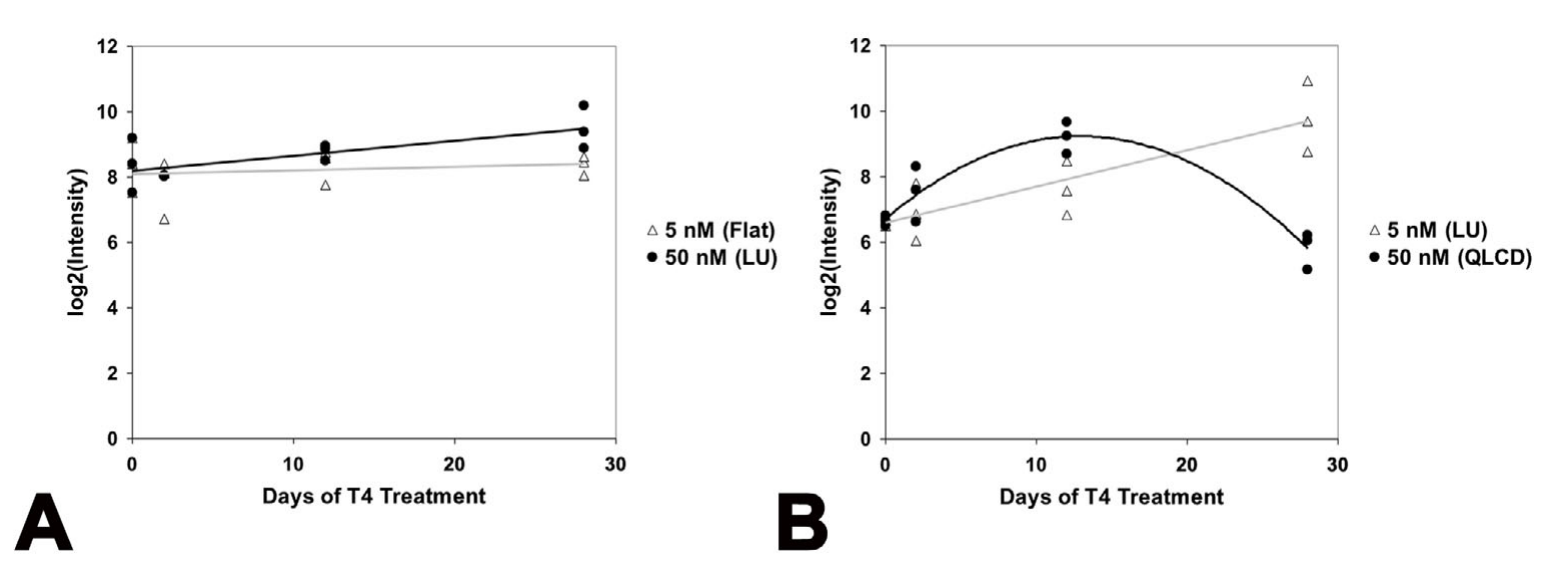
**Example regression profiles**. Example regression profiles based on microarray data from a gene that does not appreciably deviate from baseline until Day 28 in 50 nM T_4 _(A; *collagen, type VI, alpha 3*; probe-set ID: SRV_05103_a_at) and a gene that is initially up regulated in 50 nM T_4 _before being down regulated (B; *N-Myc downstream regulated gene 1*; probe-set ID: SRV_12417_at). Trend lines for the 5 nM regression analyses are gray and trend lines for the 50 nM regression analyses are black. LU = linear up and QLCD = quadratic linear concave down.

To further investigate the effect of T_4 _concentration on induced metamorphosis, we modeled mRNA abundance estimates from the 5 and 50 nM T_4 _treatments using quadratic and linear regression. The regression analyses identified 542 and 709 DEGs that changed by ≥ two-fold relative to Day 0 controls, in the 5 and 50 nM T_4 _treatments respectively [see Additional files 5, 6, 7, 8]. Given our previous analyses, we expected to observe different regression patterns (expression profiles; Figure 3) for DEGs from each treatment because metamorphic initiation timing was delayed in 5 nM T_4 _and metamorphosis was only completed in 50 nM T_4_. Indeed, most DEGs identified by the 5 nM regression analysis exhibited linear expression profiles during metamorphosis (*e.g*., linear down, LD; linear up, LU; Figure 3) while the majority of the DEGs identified by the 50 nM regression analysis exhibited curvilinear and parabolic expression profiles (*e.g*., quadratic linear convex down, QLVD; quadratic linear concave up QLCU; quadratic convex QV; quadratic concave, QC; Figure 3; see the methods for a summary of the biological interpretations of these expression profiles in the context of our experiment). Thus, biological processes known to be fundamental to tissue remodeling and/or development were identified from both T_4 _treatments, however they were statistically associated with different regression patterns (Figure 3). For example, four collagen-degrading matrix metallopeptidase (MMP) genes (*MMP13*, *MMP9*, *MMP1*) exhibited linear up regulated responses in 5 nM T_4 _and were categorized as LU. However, under 50 nM T_4_, these genes were categorized among the QC and QLCU profiles. Several genes associated with organ development (*transgelin*, *mitogen-activated protein kinase 12*, *distal-less homeo box 3*, *actin binding lim protein 1*, *collagen type VI alpha 3*, and *msh homeo box homolog 2*) were up regulated in a linear fashion in 50 nM T_4 _and were categorized as LU. In 5 nM T_4_, several of these genes (*mitogen-activated protein kinase 12*, *actin binding lim protein 1*, and *msh homeo box homolog 2*) were statistically significantly up regulated (LU and QLVU) but failed to eclipse our two-fold change criteria. A single gene (*collagen type VI alpha 3*) was not statistically significant and categorized as "Flat" in 5 nM T_4_. However, this gene did not appreciably deviate from base-line expression levels until Day 28 in 50 nM T_4 _(Figure 4A). We attribute these differences between the T_4_treatments to the delayed onset timing of metamorphosis in the 5 nM T_4 _treatment. Overall, the same generalized direction of expression was observed for 457 of 463 (99%) DEGs that were commonly identified from both T_4 _treatments (Figure 1B). The six genes (*calponin 2*, *ethylmalonic encephalopathy 1*, *3' repair exonuclease 2*, SRV_05658_a_at, SRV_09880_at, and *N-myc downstream regulated gene 1*) that seemingly exhibited opposite directions of expression in 5 and 50 nM T_4 _were all categorized the same way (LU in 5 nM T_4 _and QLCD in 50 nM T_4_). Closer inspection of these expression profiles revealed that genes classified as QLCD trend upward at the early time points before decreasing at later time points (Figure 4B). These results show that T_4 _concentration affected the shape of temporal gene expression profiles but essentially all epidermal DEGs that were identified in both T_4 _treatments were regulated in the same direction by 5 and 50 nM T_4_.

The regression analyses identified a number of DEGs that only met both of our criteria (statistically significant and ≥ two-fold change) in one of the T_4 _concentrations (5 nM, *n *= 79; 50 nM, *n *= 246; Figure 1B). Of the 79 genes unique to 5 nM T_4_, 36 (46%) were statistically significant in 50 nM T_4 _but did not eclipse our two-fold change criteria. Of the remaining 43 genes unique to 5 nM T_4_, 25 exhibited ≥ two-fold change in 50 nM T_4 _at one or more sampling times but were not statistically significant. Inspection of the 50 nM T_4 _regression patterns and fold change data associated with the 79 genes unique to 5 nM T_4 _revealed that all of these genes exhibited similar directional trends (up versus down regulation) in 5 and 50 nM T_4 _[see Additional files 9, 10]. Of the 246 genes unique to 50 nM T_4_, 96 (39%) were statistically significant in 5 nM T_4_, but failed to eclipse our two-fold change criteria. An additional 48 genes exhibited ≥ two-fold change for at least one sampling time in 5 nM T_4_, but were not statistically significant. Inspection of the 5 nM T_4 _regression patterns and fold change data associated with the 246 genes unique to 50 nM T_4 _demonstrated that 209 (85%) of these genes exhibited similar directional trends in 5 and 50 nM T_4 _[see Additional files 11, 12]. An additional 22 genes unique to 50 nM T_4 _did not exhibit > 1.5 fold changes relative to Day 0 controls until Day 28 (at which time they were differentially regulated by ≥ two-fold), suggesting that they are expressed during the terminal stages of metamorphosis [see Additional files 11, 12]. Presumably, these genes were not detected in 5 nM T_4 _because we did not sample latter time points for this concentration. These results reiterate the point that essentially all genes identified by our study were similarly, directionally expressed in the 5 and 50 nM T_4 _treatments.

To address similarity in terms of magnitude, we compared maximum fold level values for genes that exhibited QLVD and QLCU expression profiles in both T_4 _treatments (Figure 3; Table 2). We assumed that genes exhibiting these profiles had achieved maximum/minimum mRNA levels during the experiment, and thus could be reliably compared between treatments. No statistical differences were observed for fold level values of 13 QLVD and QLCU genes between the 5 and 50 nM T_4 _treatments (Wilcoxon signed rank test, *Z *= -1.293, *P *= 0.1961) [20], and the fold level values were highly correlated (Table 2; Spearman's rho = 1.00, *P *< 0.0001) [20]. Although this analysis was performed on a small subset of genes, the results suggest that mRNA abundances are similar for genes that are differentially expressed in 5 and 50 nM T_4_.

**Table 2:** Genes categorized as QLVD and QLCU in both T_4 _treatments

Gene	Probe-set ID	Fold Change 5 nM	Fold Change 50 nM	Profile
*Serpin peptidase inhibitor clade B member 10*	SRV_02418_at	3.60	3.24	QLCU
*Serpin peptidase inhibitor clade B member 2*	SRV_04787_s_at	4.23	3.76	QLCU
N/A	SRV_05867_a_at	21.70	21.44	QLCU
N/A	SRV_05889_x_at	11.05	11.01	QLCU
N/A	SRV_06225_at	24.98	26.20	QLCU
N/A	SRV_07624_x_at	9.64	9.60	QLCU
N/A	SRV_10263_at	72.28	70.70	QLCU
N/A	SRV_10508_a_at	86.94	86.10	QLCU
*Keratin 6E*	SRV_14426_at	2.97	2.95	QLCU
*WAP four-disulfide core domain 5*	SRV_05575_at	24.23	23.40	QLCU
*Keratin 4*	SRV_12057_at	327.01	417.46	QLCU
*Desmin*	SRV_01373_at	-51.18	-50.21	QLVD
N/A	SRV_01375_at	-6.03	-5.90	QLVD

N/A refers to genes that do not have established orthologies to human. Probe-set ID represents the unique identifier for each probe-set on the custom *Ambystoma *GeneChip. Fold change 5 nM refers to the maximum fold change observed in the 5 nM T_4 _treatment relative to Day 0 controls. Fold change 50 nM refers to the maximum fold change observed in the 50 nM T_4 _treatment relative to Day 0 controls. Profile refers to the expression profile for each gene obtained from regression analyses on the 5 and 50 nM T_4 _datasets. QLCU refers to the quadratic linear concave up regression profile and QLVD refers to the quadratic linear convex down regression profile.

### Bioinformatic comparison: axolotl versus Xenopus

Salamanders and anurans may express similar genes during amphibian metamorphosis. To test this idea, we compared a list of 'core' up regulated metamorphic genes from *Xenopus *[10] to DEGs identified from our study of axolotl. Of the 59 genes that were reported as differentially up regulated by ≥ 1.5 fold in limb, brain, tail, and intestine from metamorphosing *Xenopus*, 23 (39%) are represented by at least one of the 3688 probe-sets analyzed in our study. Of these, only two (*FK506 binding protein 2 *and *glutamate-cysteine ligase modifier subunit*) were identified as statistically significant and differentially expressed by ≥ two-fold in our study [see Additional files 13, 14]. *FK506 binding protein 2 *was up regulated in axolotl and *Xenopus*. However, *glutamate-cysteine ligase modifier subunit *was down regulated in axolotl and up regulated in *Xenopus*. Thus, < 5% of the 'core' DEGs that are commonly expressed among *Xenopus *tissues during metamorphosis, were identified as DEGs in our study using axolotl.

We also conducted a comprehensive bioinformatics comparison between DEGs from axolotl epidermis and DEGs from T_3 _induced *Xenopus *intestine [10]. Gene expression similarities may exist between the *Xenopus *intestine and axolotl epidermis because both organs undergo extensive extracellular remodeling that is associated with apoptosis of larval epithelial cells and the proliferation and differentiation of adult cell types. The presumptive orthologs of 111 of the 820 non-redundant DEGs from our study correspond to DEGs from *Xenopus *intestine [see Additional files 13, 14]. Of these 111 genes, 50 (45%) exhibited the same direction of differential expression in axolotl epidermis and *Xenopus *intestine. This list includes genes that are known to be associated with metamorphic developmental processes in amphibians. For example, two MMPs (*MMP9 *and *MMP13*) that are associated with extra cellular matrix turnover were up regulated in *Xenopus *intestine and axolotl epidermis. However, other genes were regulated in opposite directions. For example, *keratin 12 *and *keratin 15 *were down regulated in axolotl epidermis but up regulated in *Xenopus *intestine. These results show that there are similarities and differences in gene expression between *Xenopus *and axolotls when comparing tissues that undergo similar remodeling processes.

### Biological, technical, and statistical replication

**Figure 5:**
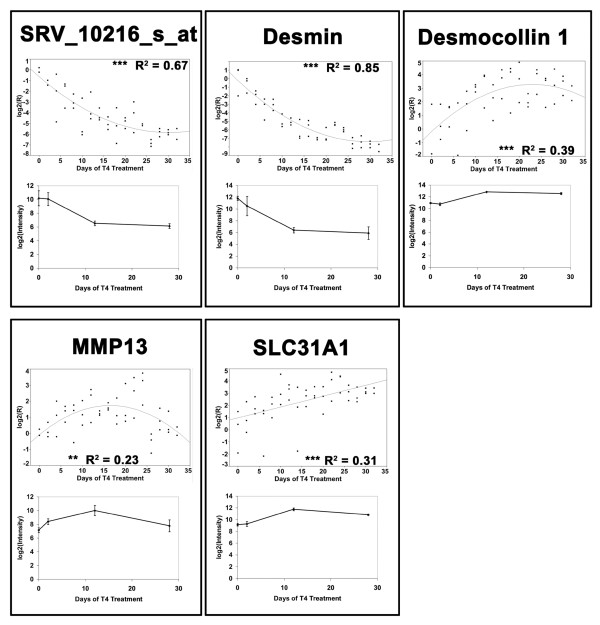
**Comparison of Q-RT-PCR and microarray data**. Comparisons of the relationships between transcript abundance and days of 50 nM T_4 _treatment as assessed in different biological samples via Q-RT-PCR (upper panels) and Affymetrix GeneChip technology (lower panels). Trend lines in the Q-RT-PCR data were obtained by linear or quadratic regression. Models with *P *< 0.01 are denoted by ** and models with *P *< 0.0001 are denoted by ***. R^2 ^refers to adjusted *R*^2^. The microarray data represent the mean of three samples ± standard deviation. MMP13 = *matrix metallopeptidase 13 *and SLC31A1 = *solute carrier family 31, member 1*.

In order to validate a subset of genes that were identified as DEGs in our microarray experiment, we conducted a second experiment in which we used quantitative real-time reverse transcriptase polymerase chain reaction (Q-RT-PCR) to generate expression profiles for five candidate genes (Table 3). These genes were chosen because they are involved in a variety of biological processes including cytoskeleton organization (*desmin*), cell-cell adhesion (*desmocollin 1*), tissue remodeling (*matrix metallopeptidase 13*), and ion transport (*solute carrier family 31 member 1*). In addition, we investigated SRV_10216_s_at in order to verify results from a gene with unknown function. Results of the regression analyses performed on the Q-RT-PCR data are presented in Figure 5 alongside plots of the analogous microarray data. Residuals from the models fit for *desmocollin 1 *and *solute carrier family 31 member 1 *exhibited significant departures from normality (Shapiro-Wilk test, *P *< 0.05). Overall, there was very good agreement between the expression profiles obtained from microarray and Q-RT-PCR analyses. The fact that the Q-RT-PCR results are biologically and technically independent of the microarray data strongly suggests that these patterns are repeatable and unlikely to be experimental or technical artifacts.

**Table 3:** Primer sequences used for Q-RT-PCR

Gene	Symbol	Probe-set ID/Direction	Sequence
*Desmin*	*DES*	SRV_01373_at_5.1	GGGCCAATTATGAAGCAA
		SRV_01373_at_3.1	CTTCGACGACTCGCTTTG
*Desmocollin 1*	*DSC1*	SRV_04693_a_at_5.1	GAGTCATTGCTCTTCCTTGG
		SRV_04693_a_at_3.1	TTTTGATGTGCTGCTCCA
*Matrix metallopeptidase 13*	*MMP13*	SRV_11423_at_5.1	TGGAAGAAGACTGCGTTGA
		SRV_11423_at_3.1	ACACTTTGAAGGCCCTTTG
*Solute carrier family 31 member 1*	*SLC31A1*	SRV_01215_a_at_5.1	TGTGATCAAACCCCAGGA
		SRV_01215_a_at_3.1	CGCGCAATCTTAAGGAAC
N/A	N/A	SRV_10216_s_at_5.1	ACATCACTGTCCCCGAAGT
		SRV_10216_s_at_3.1	GGTCGGGGTCTGTTCAAT

N/A refers to a gene that does not have an established orthology with human. Probe-set ID represents the unique identifier for each probe-set on the custom *Ambystoma *GeneChip. Forward and reverse primers are denoted by the extensions 5.1 and 3.1 respectively.

Previously, we used stringent statistical criteria (one-way ANOVA, FDR = 0.001, and ≥ two-fold change) to identify 123 annotated genes that exhibited robust responses to 50 nM T_4 _[11]. In that study, we focused on the potential of several keratin loci to serve as biomarkers of early metamorphic changes that precede changes in gross morphology. In this study, we used less stringent criteria to identify DEGs and more fully explore temporal gene expression responses when T_4 _concentration is varied. Of the 123 DEGs previously identified in the epidermis of metamorphosing axolotls, 116 genes were statistically significant and differentially regulated by ≥ two-fold in the 50 nM regression analysis. Of these, 91 (78%) were statistically significant and differentially expressed by ≥ two-fold in the 5 nM regression analysis. Only one of these 91 genes was expressed in opposite directions between the T_4 _treatments (*3' repair exonuclease 2*). However this gene was classified as LU in 5 nM T_4 _and QLCD in 50 nM T_4_, and represents another example of a transiently up regulated gene that was categorized as QLCD [see Additional files 7, 8]. The 25 genes identified in 50 nM T_4 _but not 5 nM T_4 _(Table 4) may function in late stage metamorphic processes that were only attained within 28 days under 50 nM T_4_. For example, *keratin 17 *is known to be a marker of proliferating basal epidermal stem cells in mammals [21]; this gene may be expressed late during metamorphosis in terminal cell populations of axolotl epidermis that give rise to adult epithelial cells. Other genes associated with tissue stress, injury, and immune function (*ferritin heavy polypeptide 1*, *ras-related C3 botulinum toxin substrate 2*, and *cathespin S*) also appear to be late response genes although we can't rule out the possibility that these genes may be differentially expressed as a toxic response to 50 nM T_4_. The majority of the DEGs identified previously using 50 nM T_4 _and very strict statistical criteria were similarly identified using 5 nM T_4 _and different statistical methods/criteria. These findings further emphasize that the metamorphic gene expression programs of *A. mexicanum *are similar even when TH concentration is varied by an order of magnitude.

**Table 4:** Genes previously identified using 50 nM T_4 _that were not identified using 5 nM T_4_

Gene	Probe-set ID	Profile
*Actin alpha 2*	SRV_01045_a_at	LU
*Actin alpha 2*	SRV_11152_at	LU
*Cathepsin S*	SRV_02072_at	QLCD
*Chormosome 20 open reading frame 92*	SRV_05090_at	QLVD
*Chromosome 21 open reading frame 33*	SRV_02285_at	QLCD
*Cytochrome C oxidase subunit VIB polypeptide 1*	SRV_01220_at	QLCD
*Cytochrome P450 family 2 subfamily c polypeptide 8*	SRV_00512_at	QLCD
*EPH receptor A1*	SRV_12195_at	QC
*Eukaryotic translation initiation factor 4E binding protein 3*	SRV_01958_at	QV
*Ferritin heavy polypeptide 1*	SRV_01294_at	QLCD
*Glutaredoxin*	SRV_01306_at	LD
*Glycine C-acetyltransferase*	SRV_03491_a_at	LU
*High-mobility group 20 A*	SRV_04181_at	QLVD
*Hypothetical protein FLJ11151*	SRV_04229_at	QLVU
*Keratin 17*	SRV_00389_a_at	QLCD
*LSM10 U7 small nuclear RNA associated*	SRV_05010_at	QLVD
*Mediator of RNA polymerase II transcription subunit 31 homolog*	SRV_03843_a_at	QLVD
*Myosin heavy polypeptide 11*	SRV_11441_at	LU
*Myosin light polypeptide 9*	SRV_12418_at	LU
*Nick-associated protein 1*	SRV_03398_at	LU
*Non-metastatic cells 7*	SRV_03369_at	QLVD
*P450 oxidoreductase*	SRV_00555_at	LU
*Phosphoglucomutase 2*	SRV_04215_at	QLVU
*Ras-related C3 botulinum toxin substrate 2*	SRV_01633_at	QLCD
*Ubiquitin specific peptidase 10*	SRV_02461_at	QLCU

Probe-set ID represents the unique identifier for each probe-set on the custom *Ambystoma *GeneChip. Profile refers to the regression profile obtained from the regression analysis of the 50 nM T_4 _dataset. LU = linear up, QLCD = quadratic linear concave down, QC = quadratic concave, QV = quadratic convex, QLVD = quadratic linear convex down, LD = linear down, QLCU = quadratic linear concave up, and QLVU = quadratic linear convex up.

## Discussion

Paedomorphic Mexican axolotls can be induced to undergo metamorphosis by administering TH. We found that axolotls initiate metamorphosis at least one week earlier in 50 nM versus 5 nM T_4 _and complete morphological transformations in 28 days. The lower 5 nM T_4 _concentration was sufficient to induce metamorphosis but the initiation timing was delayed and this proportionally delayed the time to complete metamorphosis. The same sequence of morphological changes was observed between T_4 _treatments and the majority of DEGs were identified in both T_4 _treatments, although their expression profiles were temporally shifted. Nearly all DEGs exhibited similar directional trends between treatments, and the subset of genes that were directly compared between the T_4 _treatments exhibited similar relative abundances. Our results show extremely similar changes in gene expression and morphology in the axolotl when varying T_4 _by an order of magnitude. This is an interesting result because T_4 _concentrations within this range are toxic to anurans and are known to affect tissue-specific abundances of transcription factors that regulate metamorphic gene expression programs [16]. Below, we discuss the axolotl's precise transcriptional response to the range of T_4 _concentrations examined in this study. We then discuss the epidermal gene expression program of the axolotl, noting gene expression similarities and differences between salamander and anuran metamorphosis.

TH levels are known to increase in larval amphibians as they mature and reach maximal levels during metamorphic climax. When the concentration of TH reaches a critical intracellular level, transcriptional changes are initiated that bring about new patterns of development. Because the TH concentration required to alter transcription is cell-specific, tissues are often described as having different sensitivities to TH. The sensitivity of cells to TH involves multiple factors that affect the intracellular concentration of TH and the ability of TH to affect transcription, which is determined in part by the number of nuclear TH binding sites (TRs) [22]. Mexican axolotls have functional TRs [23], but TH levels are apparently too low to initiate metamorphosis [24]. Direct hypothalamic application of T_4_, using a dose that is insufficient to initiate metamorphosis via intraperitoneal injection, is sufficient to initiate metamorphosis in the axolotl [7] and related paedomorphic tiger salamanders [25]. Thus, axolotls are capable of synthesizing TH in sufficient quantities to initiate and complete metamorphosis. However, the pituitary doesn't release a sufficient amount of thyrotropin to trigger the metamorphic process [24]. Axolotl epidermis can be stimulated to initiate metamorphic changes *in vitro*, in isolation of endogenously synthesized TH [26]. Thus, the metamorphic timing delay that we observed between the 5 and 50 nM T_4 _treatments probably reflects the time required to autonomously activate gene expression within TH responsive cells of the epidermis and the time required to stimulate the HPT axis. Rosenkilde [27] showed that this latency period is TH concentration dependent and above a critical dose (37.5 nM T_3_) there is no variation in latency. After accounting for an estimated one-week difference in the initiation timing of metamorphosis between the T_4 _treatments, there was not a difference in the length of the metamorphic period. Thus, endogenous TH levels were functionally, if not quantitatively similar between 5 and 50 nM T_4 _treated axolotls after metamorphosis was initiated. This idea is also supported by the precise gene expression response that we observed between the T_4 _treatments: essentially all of the genes were expressed in the same direction, and a subset of genes that could be reliably compared showed the same magnitude of gene expression.

The precision of the transcriptional response between T_4 _treatments indicates that axolotls are surprisingly tolerant to T_4 _levels that dramatically affect anuran mortality and gene expression. Others before us have also noted the tolerance of axolotls to high levels of T_4 _[27,28]. Because anuran metamorphosis involves a more extensive and integrated set of remodeling events that are accomplished over a shorter time frame, there may be greater overlap in the sensitivities of cells to TH among tissues that causes metamorphic remodeling events to occur out of sequence. The fact that salamander metamorphosis encompasses fewer morphological changes and that many of the changes are not as integrated (hindlimb development occurs months before tail metamorphosis) may explain why axolotls are so tolerant to high T_4 _concentrations. However, failure to observe an increase in *thyroid hormone receptor beta *transcription in axolotl suggests there may be fundamental regulatory differences between anuran and salamander metamorphosis.

The larval epidermis of axolotls is extensively remodeled during T_4 _induced metamorphosis [29-31]. Application of TH to paedomorphic axolotls induces many of the same epidermal changes that occur during natural and induced metamorphosis in anurans, including apoptosis of larval cells, proliferation of adult cell types, and epidermal keratinization. Our results show that TH induces a diversity of transcriptional changes that are associated with specific remodeling processes. We observed significant gene expression changes between the T_4 _treatments at Day 2, prior to observable morphological changes at the whole-organism level. Most of these genes were up regulated in the higher T_4 _concentration relative to the lower T_4 _concentration. Day 2 gene expression changes may reflect direct transcriptional activation via the binding of exogenous TH to TRs, which are functional in axolotls [23]. For example, the human ortholog of *phosphoenolpyruvate carboxykinase 1*, a primary target for transcriptional regulation of gluconeogenesis, is known to have a thyroid hormone response element [32]. Early up regulation of *phosphoenolpyruvate carboxykinase 1*, as well as *fructose 1,6 bisphosphotase, glucose 6 phosphate dehydrogenase*, and *70 kD heat shock protein 5 *at Day 12, indicates a biochemical response at the cellular level that includes activation of key regulatory enzymes of the gluconeogenic pathway. This is an interesting finding for the epidermis because such responses are generally associated with hepatic cell functions. Several other interesting gene expression changes were detected at Day 2. These include *ATP binding cassette, subfamily B, member 4*, and *transglutaminase *1. ATP binding cassette family genes are up regulated in mammals during epidermal lipid reorganization and keratinocyte differentiation [33], and *transglutaminase *1 encodes an enzyme that functions in the formation of the cross-linked, cornified envelop of keratinocytes [34]. The early expression of these genes is curious because keratinization is assumed to be a terminal differentiation event in the metamorphosis of amphibian epidermis. Our results suggest that the process of keratinization is initiated very early. As a final example, two proteins that are specific to the mammalian inner ear were identified as significantly down regulated: *otogelin *and *otoancorin*. The head epidermis of the axolotl contains mechanoreceptors that are homologous to hair cells of the mammalian ear [35]. Our results suggest remodeling of these and other neural components in the axolotl skin at metamorphosis. There are many additional examples that could be highlighted from our gene lists that have not been previously discussed within the context of amphibian metamorphosis.

The most gene expression changes and the greatest changes in transcript abundances were observed at Day 12 in 50 nM T_4_. For example, *keratin 14*, a prototypical marker of proliferating keratinocytes in mammals [36], was up regulated 1146 fold in 50 nM T_4_. This also marks the time of the greatest morphological remodeling. After this time, gene expression levels of many genes decreased. Thus, as has been described in anurans, many gene expression changes in axolotl are transient, increasing initially and then decreasing. For example, apoptosis is activated and terminated during anuran [37] and salamander [38] metamorphosis to regulate the death and replacement of larval epithelial cells. When statistically significant genes were analyzed in the absence of a two-fold change criterion, genes that were transiently up regulated (*i.e*., exhibited QC profiles) were statistically associated with apoptosis and proteolysis functional ontologies (data not shown). As another example of the similarities between the metamorphic gene expression changes that occur in the epidermis of frogs and salamanders, we identified three distinct probe-sets with established orthologies to human *uromodulin *that are dramatically down regulated in the epidermis of metamorphosing *A. mexicanum*. Furlow et al. [39] have observed analogous results in *X. laevis *and have shown that *Xenopus uromodulin *orthologs are exclusively expressed in the apical cells of the larval epidermis. These and other genes that are similarly expressed between urodeles and anurans will provide useful biomarkers for comparative studies of metamorphosis between these two groups.

Our informatics comparison between DEGs identified from axolotl epidermis and *Xenopus *intestine identified > 100 genes that are commonly expressed in these organs during metamorphic remodeling. However, over half of these genes were differentially expressed in opposite directions in axolotl and *Xenopus*. For example, several genes associated with immune function (*CD74 antigen, chemokine ligand 5, interferon regulatory factor 1, proteasome beta subunit 9*, and *class I-related major histocompatability complex*) were down regulated in axolotl epidermis and up regulated in *Xenopus *intestine. This is not too surprising because it is well established that the axolotl immune system is fundamentally different from that of other vertebrates, including *Xenopus *[40]. Additionally, genes expressed in opposite directions in these comparisons may reflect fundamental differences that exist between intestinal and epidermal remodeling. Genes that exhibited similar transcriptional patterns between *Xenopus *intestine and axolotl epidermis were associated with many different functions. For example, *DNA methyltransferase 1 *and *17-beta hydroxysteroid dehydrogenase 8 *were down regulated in axolotl epidermis and *Xenopus *intestine during induced metamorphosis. In mammals, *DNA methyltransferase 1 *functions to maintain DNA methylation patterns that influence gene transcription [41] and *17-beta hydroxysteroid dehydrogenase 8 *preferentially inactivates androgens and estrogens, [42,43]. This later example suggests a transcriptional response to increase gonadal steroid hormone levels during epithelial remodeling in amphibians. As a final example, a presumptive ortholog to human *keratin 24 *(SRV_13498_s_at) that was up regulated by > 1000 fold in axolotls exposed to 50 nM T_4 _was also up regulated in *Xenopus *intestine, albeit by a comparatively modest four-fold increase. These comparisons emphasize similarities and differences in gene expression during metamorphic epithelial tissue remodeling in anurans and salamanders.

## Conclusion

Recent microarray analyses of anurans and salamanders show that amphibian metamorphosis involves thousands of gene expression changes, involving many biological processes that have previously received little attention [9,10]. Our results show similarities and differences in the metamorphic transcriptional programs of anurans and salamanders. We expected to identify similarly expressed genes because epidermis was included in anuran tissue preparations that were used for microarray analysis, and because tissue remodeling that occurs during metamorphosis appears to involve some evolutionarily conserved biological processes. We also expected to observe transcriptional differences because anuran and salamander lineages diverged > 300 million years ago. Our results suggest that amphibian metamorphosis cannot be fully understood from the study of a few anuran species. We show here that axolotls offer several advantages (inducible metamorphosis, robust transcriptional response, less complex integration of remodeling events) that can be exploited to provide complementary and novel perspectives on amphibian metamorphosis.

## Methods

### Study animals for microarray analyses

All procedures were conducted in accordance with the University of Kentucky Animal Care and Use Guidelines (IACUC #00609L2003). Salamanders (*A. mexicanum*) were obtained from a single genetic cross, using adults from an inbred strain. Embryos and larvae were reared individually at 20–22 C in 40% Holtfreter's solution. After hatching, larvae were fed freshly hatched brine shrimp (*Artemia *sp., Brine Shrimp Direct, Ogden, UT) napuli until they were large enough (3 weeks) to eat California blackworms (*Lumbriculus *sp., J.F. Enterprises, Oakdale, CA). At approximately eight months of age, 30 salamanders were randomly assigned to each of 10 different treatments and reared in 40% Holtfreter's solution with or without T_4_, (Sigma T2376, St. Louis, MO). One hundred μM stock T_4 _solutions were made as described in Page et al. [11]. Five and 50 nM T_4 _were made fresh for each water change by mixing 2.5 or 25 mL of 100 μM stock with 40% Holfreter's solution to a final volume of 50 L. Water was changed every third day.

Skin tissue was collected from salamanders following 0, 2, 12, and 28 days of T_4 _treatment. These time points were sampled to test for early gene expression changes that might precede morphological metamorphosis, and because 28 days is a sufficient period for complete metamorphosis of 50 nM T_4 _induced *A. mexicanum *[11]. To obtain tissue, salamanders were anesthetized in 0.01% benzocaine (Sigma, St. Louis, MO) and ≈ 1 cm^2 ^of skin tissue was removed from the top of the head.

### RNA isolation

Total RNA was extracted for each tissue sample with TRIzol (Invitrogen, Carlsbad, CA) according to the manufacturer's protocol; additionally, RNA preparations were further purified using a Qiagen RNeasy mini column (Qiagen, Valencia, CA). UV spectrophotometry and a 2100 Agilent Bioanalyzer (Agilent Technologies, Santa Clara, CA) were used to quantify and qualify RNA preparations. Three high quality RNA isolations from each treatment and sampling time combination were used to make individual-specific pools of biotin labeled cRNA probes. Each of the 30 pools was subsequently hybridized to an independent GeneChip. The University of Kentucky Microarray Core Facility generated cRNA probes and performed hybridizations according to standard Affymetrix protocols.

### Microarray platform

A custom *Ambystoma *Affymetrix (Santa Clara, CA) GeneChip was designed from curated expressed sequence tag assemblies for *A. mexicanum *and *A. tigrinum *[44,45]. The array contains 4844 probe-sets, 254 of which are controls or replicate probe-sets. Detailed descriptions of this microarray platform can be found in Page et al. [11] and Monaghan et al. [46].

### Quality control and low-level analyses

We used the Bioconductor package affy [47] that is available for the statistical programming environment R [48] to perform a variety of quality control and preprocessing procedures at the individual probe level [49,50]. These procedures included: 1) generating matrices of M versus A plots for all replicate GeneChips (*n *= 3 GeneChips for 10 treatment/sampling time combinations), 2) investigating measures of central tendency, measures of dispersion, and the distributions of all 30 GeneChips via boxplots and histograms, 3) viewing images of the log_2_(intensity) values for each GeneChip to check for spatial artifacts, and 4) viewing an RNA degradation plot [50] that allows for visualization of the 3' RNA labeling bias across all GeneChips simultaneously. In addition, we used ArrayAssist Lite software (Stratagene, La Jolla, CA) to assess several quality control measures that are recommended by Affymetrix such as average background (minimum = 48.36, maximum = 59.28, *n *= 30) scale factors (minimum = 0.404, maximum = 0.629, *n *= 30), and percent present (minimum = 81.5, maximum = 86.5, *n *= 30). Next, we processed our data by implementing the robust multi-array average (RMA) algorithm of Irizarry et al. [51].

### Assessment of GeneChip precision

To obtain estimates of between GeneChip repeatability, we generated correlation matrices for the hybridization intensities across all probe-sets among replicate GeneChips. Very high and consistent mean *r*-values were calculated for each of the 10 treatment by sampling time combinations (range of mean *r *± standard error = 0.966 ± 0.002 to 0.986 ± 0.001). These results demonstrate that we were able to obtain a high level of repeatability between replicate GeneChips. Our data are MIAME compliant and raw data files can be obtained at Sal-Site [45,52].

### Data filtering

Microarray platforms may not accurately or precisely quantify genes with low intensity values [53,54]. Because low intensity genes contribute to the multiple testing problem that is inherent to all microarray studies, we filtered probe-sets whose mean expression values across all GeneChips (*n *= 30 per gene) were smaller than or equal to the mean of the lowest quartiles (25^th ^percentiles) across all GeneChips (*n *= 30, mean = 6.53, standard deviation = 0.04; data presented on a log_2 _scale). Upon performing this filtration step, 3688 probe-sets were available for significance testing. We then performed PCA on the centered and scaled data from these probe-sets. This analysis allowed us to visualize the relationships between GeneChips within and across treatments.

### Temporal gene expression in the absence of T_4_

We investigated whether genes exhibited differential expression as a function of time in the absence of T_4 _(control animals sampled at Days 0, 2, 12, and 28) via linear and quadratic regression [55]. We corrected for multiple testing by evaluating α_0 _according to the algorithm of Benjamini and Hochberg [56] with a FDR of 0.05. α_1 _was set to 0.05.

### Detecting and classifying DEGs

We conducted three analyses to investigate the effect of T_4 _on epidermal gene expression. For the first analysis, we used limma [57,58] to identify genes that were differentially expressed as a function of T_4 _concentration. The limma package couples linear models with an empirical Bayes methodology to generate moderated *t*-statistics for each contrast of interest. This approach has the same effect as shrinking the variance towards a pooled estimate and thus reduces the probability of large test statistics arising due to underestimations of the sample variances. Operating limma requires the specification of two matrices. The first is a design matrix in which the rows represent arrays and the columns represent coefficients in the linear model. The second is a contrast matrix in which the columns represent contrasts of interest and the rows represent coefficients in the linear model. For this analysis, the design matrix specified a coefficient for each unique treatment by sampling time combination (10 coefficients) and the contrast matrix specified the calculation of contrasts between the two T_4 _concentrations (5 and 50 nM) at each of the non-zero sampling times (Days: 2, 12, and 28). In addition to moderated *t*-statistics, limma also generates moderated *F*-statistics. These moderated *F*-statistics test the null hypothesis that no differences exist among any of the contrasts specified by a given contrast matrix. A FDR correction [56] of 0.05 was applied to the *P*-values associated with the moderated *F*-statistics of the contrast matrix. In order to further reduce the number of false positives, we required that all "identified" DEGs be differentially regulated by ≥ two-fold at one or more of the contrasted time points.

The last two analyses were conducted using the regression-based approach of Liu et al. [55] to detect genes that exhibit differential expression as a function of days of T_4 _treatment. This approach also classifies DEGs into nine categories based on their temporal expression profiles as determined via linear and quadratic regression. In the context of our experiment, these profiles have specific biological interpretations. However, exceptions to these interpretations exist (see results for examples). In general, genes that exhibited LD, LU, QLCD, and QLVU expression profiles were still actively undergoing changes in their expression levels when the study was terminated. In contrast, genes that exhibited QLVD and QLCU expression profiles underwent down and up regulation respectively but reached steady state expression levels before the experiment was terminated. Finally, genes that exhibited QC and QV expression profiles were transiently up and down regulated respectively before returning to baseline expression levels. Null results are described by the 'Flat' category. Separate analyses were conducted for the 5 and 50 nM datasets with α_0 _evaluated at a FDR of 0.05 according to the algorithm of Benjamini and Hochberg [56] and α_1 _= 0.05. DEGs were required to exhibit ≥ two-fold changes relative to Day 0 controls at one or more sampling times before they were categorized as "identified".

### Over representation analyses for genes with established orthologies

Biological process gene ontology categories that are over represented in our lists of DEGs (statistically significant and ≥ two-fold change) were identified using the Database for Annotation Visualization and Integrated Discovery (DAVID) [59]. In all analyses, the 3085 probe-sets on the *Ambystoma *GeneChip with established orthologies were used as the background for generating expected values. The EASE threshold was always set to 0.05, and the count threshold was always set to two.

### Bioinformatic comparison with Xenopus

In a recent microarray study, Buchholz et al. [10] presented "a core set of up regulated genes". These genes have been identified as up regulated in response to T_3 _treatment by ≥ 1.5 fold in every tissue that has been examined in metamorphosing *X. laevis *via microarray analysis (limb, brain, tail, and intestine) [9,10]. We determined the orthologies of these genes to human as described in Page et al. [11] and Monaghan et al. [46]. We then identified genes listed by Buchholz et al. [10] that were also differentially expressed in our experiment. The same approach was used to compare our gene lists to the 2340 DEGs identified by Buchholz et al. [10] from the intestine of metamorphosing *X. laevis*.

### Biologically and technically independent verification

We conducted a second experiment using Q-RT-PCR to investigate the temporal expression patterns of five genes identified as differentially expressed by microarray analysis (Table 3). Animals used in our second experiment were raised as described for the animals used in the microarray experiment, with the exception that T_4 _treatment (50 nM) was initiated at 120 days post-fertilization. Tissue samples from two or three individuals were collected as described above beginning on Day 0 (prior to initiating T_4 _treatment) and were collected every two days for 32 days (*i.e*., Day 0, Day 2, Day 4... Day 32).

### Q-RT-PCR

Total RNA was extracted from integument as described for the microarray experiment with the exception that all samples were treated with RNase-Free DNase Sets (Qiagen, Valencia, CA) according to the manufacturer's protocol. For each sample, the Bio-Rad iScript cDNA synthesis kit (Hercules, CA) was used to synthesize cDNA from 1 μg total RNA. Primers (Table 3) were designed using Primer3 [60], and design was targeted to the same gene regions that are covered by Affymetrix probe-sets. All PCRs were 25 μL reactions that contained: cDNA template corresponding to 10 ng total RNA, 41 ng forward and reverse primers, and iQ SYBR-Green real-time PCR mix (Bio-Rad, Hercules, CA). Reaction conditions were as follows: 10 minutes at 50 C, five minutes at 95 C, 45 cycles of 10 seconds at 95 C followed by 30 seconds at 55 C, one minute at 95 C, and one minute at 55 C. Melting curve analysis was used to ensure the amplification of a single product for each reaction. All reactions were run in 96 well plates and blocked by sampling time (*i.e*., each of the 17 sampling times was equally represented for each gene on each plate). PCRs were performed using a Bio-Rad iCycler iQ Multi-Color Real Time PCR Detection System (Hercules, CA). All plates contained template free controls [61]. Primer efficiencies were estimated via linear regression and relative expression ratios (R) were calculated according to Pfaffl [62]. All expression ratios are relative to the average of the Day 0 animals, and normalized to *transcriptional intermediary factor 1 *(probe-set ID: L_s_at). This gene was selected as a control because it had an extremely small standard deviation across all treatment regimes in the microarray experiment (*n *= 30, mean = 14.44, standard deviation = 0.03; data presented on a log_2 _scale).

### Statistical Analysis of the Q-RT-PCR Data

Log_2 _transformed R-values for each gene were analyzed separately via linear and quadratic regression models in which days of T_4 _treatment was the predictor variable. These analyses were carried out using JMP, Version 5 (SAS Institute, Cary, NC). We decided whether to use a linear or quadratic model for a given gene via forward selection [20]. In short, quadratic models were accepted when the polynomial terms were significant (*P *< 0.05) and resulted in an increase in the proportion of variation in the data explained by the model (adjusted *R*^2^) of ≥ five percent relative to the linear model. The residuals of all models were inspected graphically. In addition, the residuals of all models were checked for normality. In cases where the assumption of normality was violated, regression analyses were run to obtain equations that describe the response of these genes to T_4 _as a function of time. However, such analyses were conducted with the understanding that a strict hypothesis testing interpretation could prove problematic.

## Authors' contributions

RBP carried out statistical and informatic analyses, conducted the Q-RT-PCR experiment, and helped draft the paper. SRV conceived the research project, provided general oversight for the project, and participated in drafting the manuscript. AKS reared the animals associated with the microarray experiment, collected tissue, and extracted RNA. JJS participated in rearing the animals associated with the microarray experiment and contributed to the statistical analyses that were conducted. SP conducted the informatic analyses used to determine the presumptive orthologies of Xenopus genes to human. CKB helped conceive the project, participated in coordinating the project, and provided critical reviews of the manuscript. All authors have read and approved the final version of this manuscript.

## Supplementary Material

Additional file 1 Analysis of the effect of time in the absence of T_4_. Excel table containing statistical and informatic values associated with the analysis of the effect of time in the absence of T_4_. Click here for file

Additional file 2 Description of the effect of time in the absence of T_4_. Word document containing descriptions of the column headers in Additional file 1. Click here for file

Additional file 3 DEGs identified by contrasting the T_4 _concentrations at each non-zero time point. Excel table containing statistical and informatic values for the DEGs identified by contrasting the 5 and 50 nM T_4 _treatments at Days 2, 12, and 28 and imposing fold change criteria. Click here for file

Additional file 4 Description of the DEGs identified by contrasting the T_4 _concentrations at each non-zero time point. Word document containing descriptions of the column headers in Additional file 3. Click here for file

Additional file 5 DEGs identified by the 5 nM regression analysis. Excel table containing statistical and informatic values for the DEGs identified by conducting linear and quadratic regression on the 5 nM dataset and imposing fold change criteria. Click here for file

Additional file 6 Description of the DEGs identified by the 5 nM regression analysis. Word document containing descriptions of the column headers in Additional file 5. Click here for file

Additional file 7 DEGs identified by the 50 nM regression analysis. Excel table containing statistical and informatic values for the DEGs identified by conducting linear and quadratic regression on the 50 nM dataset and imposing fold change criteria. Click here for file

Additional file 8 Description of the DEGs identified by the 50 nM regression analysis. Word document containing descriptions of the column headers in Additional file 7. Click here for file

Additional file 9 The 79 genes unique to the 5 nM regression analysis. Excel table containing statistical and informatic values for the 79 DEGs identified by the 5 nM regression analysis and fold change criteria that were not identified by the 50 nM regression analysis. Click here for file

Additional file 10 Description of the 79 genes unique to the 5 nM regression analysis. Word document containing descriptions of the column headers found in Additional file 9. Click here for file

Additional file 11 The 246 genes unique to the 50 nM regression analysis. Excel table containing statistical and informatic values for the 246 DEGs identified by the 50 nM regression analysis and fold change criteria that were not identified by the 5 nM regression analysis. Click here for file

Additional file 12 Description of the 246 genes unique to the 50 nM regression analysis. Word document containing descriptions of the column headers found in Additional file 11. Click here for file

Additional file 13 The 111 genes identified as differentially expressed in axolotl epidermis and *Xenopus *intestine. Excel table containing statistical and informatic values for the genes identified as differentially expressed in axolotl epidermis and *Xenopus *intestine. Click here for file

Additional file 14 Description of the 111 genes identified as differentially expressed in axolotl epidermis and *Xenopus *intestine. Word document containing descriptions of the column headers in Additional file 13. Click here for file
