# Supplementary material for: Effect of thyroid hormone concentration on the transcriptional response underlying induced metamorphosis in the Mexican axolotl (Ambystoma)
Source: BMC Genomics. 2008 Feb 11;9:78. doi: 10.1186/1471-2164-9-78 (PMC2262897; doi:10.1186/1471-2164-9-78)
Supplement: Additional file 2 — Description of the effect of time in the absence of T4. Word document containing descriptions of the column headers in Additional file 1. [file 1471-2164-9-78-S2.doc]

Analysis of the effect of time in the absence of T_4_.

Column A: Unique probe-set ID for probe-sets on the custom *Ambystoma* GeneChip

Column B: The rank a given gene’s *P*-value (1 = smallest *P*-value)

Column C: The FDR adjusted significance threshold against which the overall *P*-values are assessed

Column D: *P*-value associated with the overall model fit to a given probe-set

Column E: *P*-value associated with the quadratic term in a model fit to a given probe-set

Column F: *P*-value associated with the linear term in a model fit to a given probe-set

Column G: The intercept of a model fit to a given probe-set

Column H: Coefficient for the linear term in a model fit to a given probe-set

Column I: Coefficient for the quadratic term in a model fit to a given probe-set

Column J: Name of the presumptive human ortholog

Column K: The expression pattern observed for a given probe-set

Column L: Logical statement describing whether the model fit to a given probe-set is statistically significant upon adjusting the FDR of α_0_ to 0.05 (yes = significant)
