# Supplementary material for: Effect of thyroid hormone concentration on the transcriptional response underlying induced metamorphosis in the Mexican axolotl (Ambystoma)
Source: BMC Genomics. 2008 Feb 11;9:78. doi: 10.1186/1471-2164-9-78 (PMC2262897; doi:10.1186/1471-2164-9-78)
Supplement: Additional file 4 — Description of the DEGs identified by contrasting the T4 concentrations at each non-zero time point. Word document containing descriptions of the column headers in Additional file 3. [file 1471-2164-9-78-S4.doc]

Genes that were statistically significant and differentially regulated by ≥ two-fold in the analysis of the effect of T_4_ concentration at specific non-zero time points. Sal-Site is an *Ambystoma* data repository that is publicly accessible on the World Wide Web (www.ambystoma.org).

Column A: Unique probe-set ID for probe-sets on the custom *Ambystoma* GeneChip

Column B: Sal-Site contig name

Column C: Sal-Site contig identifier

Column D: The best human BLASTX hit to a salamander contig query

Column E: E-value for the BLASTX search described for the previous column

Column F: RefSeq identifer for human hits associated with BLASTX searches

Column G: Name of the human hit associated with BLASTX searches

Column H: Entrez gene identifier of the best human hit associated with BLASTX searches

Column I: URL associated with the Entrez gene identifier mentioned for the previous column

Column J: Moderated *F-*statistic associated with the contrast matrix

Column K: Probability of observing the moderated *F-*statistic

Column L: Moderated *t-*statistic associated with the contrast performed for Day 2

Column M: Moderated *t*-statistic associated with the contrast performed for Day 12

Column N: Moderated *t*-statistic associated with the contrast performed for Day 28

Column O: Probability of observing the moderated *t*-statistic for the contrast at Day 2

Column P: Probability of observing the moderated *t*-statistic for the contrast at Day 12

Column Q: Probability of observing the moderated *t*-statistic for the contrast at Day 28

Columns R, T, and V: Multiple testing corrections associated with each of the three contrasts. 0 = no change, -1 = down-regulated in 50 nM relative to 5 nM, 1 = up-regulated in 50 nM relative to 5 nM

Columns S, U, and W: Logical statements (for sorting) indicating whether a contrast corresponding to a particular sampling time is significant irrespective of the direction of significance.

Column X: Logical statement indicating whether any of the contrasts performed are statistically significant upon implementing the multiple testing correction. TRUE = no significant contrasts, FALSE = at least one significant contrast

Columns Y-AD: Back-transformed (raw scale) mean values (*n* = 3 per group) for each non-control treatment by sampling time group.

Columns AE-AG: Back-transformed ratios (raw scale) for each of the contrasts performed. 50 nM data are in the numerators and 5 nM data are in the denominators. Thus, values equal to one represent identical expression levels in the two concentration treatments. Values > 1 describe genes that are up-regulated in the 50 nM samples relative to the 5 nM samples. Values < 1 describe genes that are down-regulated in the 50 nM damples relative to the 5 nM samples.
