# Supplementary material for: Effect of thyroid hormone concentration on the transcriptional response underlying induced metamorphosis in the Mexican axolotl (Ambystoma)
Source: BMC Genomics. 2008 Feb 11;9:78. doi: 10.1186/1471-2164-9-78 (PMC2262897; doi:10.1186/1471-2164-9-78)
Supplement: Additional file 6 — Description of the DEGs identified by the 5 nM regression analysis. Word document containing descriptions of the column headers in Additional file 5. [file 1471-2164-9-78-S6.doc]

Genes that were statistically significant and differentially regulated by ≥ two-fold in the 5 nM regression analysis. Sal-Site is an *Ambystoma* data repository that is publicly accessible on the World Wide Web (www.ambystoma.org).

Column A: Unique probe-set ID for probe-sets on the custom *Ambystoma* GeneChip

Column B: Sal-Site contig name

Column C: Sal-Site contig identifier

Column D: The best human BLASTX hit to a salamander contig query

Column E: E-value for the BLASTX search described for the previous column

Column F: RefSeq identifer for human hits associated with BLASTX searches

Column G: Name of the human hit associated with BLASTX searches

Column H: Entrez gene identifier of the best human hit associated with BLASTX searches

Column I: URL associated with the Entrez gene identifier mentioned for previous the column

Column J: The rank of a given probe-set based on the over-all *P*-value (1 corresponds to the smallest *P*-value)

Column K: The FDR adjusted significance threshold against which the overall *P*-values are assessed

Column L: *P*-value associated with the overall model fit to a given probe-set

Column M: *P*-value associated with the quadratic term in a model fit to a given probe-set

Column N: *P*-value associated with the linear term in a model fit to a given probe-set

Column O: The intercept of a model fit to a given probe-set

Column P: Coefficient for the linear term in a model fit to a given probe-set

Column Q: Coefficient for the quadratic term in a model fit to a given probe-set

Column R: The expression pattern observed for a given probe-set

Column S: Logical statement describing whether the model fit to a given probe-set is statistically significant upon adjusting the FDR of α_0_ to 0.05 (yes = significant)

Columns T-W: Back-transformed (raw scale) mean values for Day 0 controls and the non-control treatment by sampling time groups.

Columns X-Z: Back-transformed (raw scale) fold change values for each non-control treatment by sampling time. The non-control is in the numerator and Day 0 is in the denominator. Values of 1 indicate that expression is identical to Day 0 values > 1 indicate up-regulation relative to Day 0 and values < 1 indicate down regulation relative to Day 0.
