# Supplementary material for: Effect of thyroid hormone concentration on the transcriptional response underlying induced metamorphosis in the Mexican axolotl (Ambystoma)
Source: BMC Genomics. 2008 Feb 11;9:78. doi: 10.1186/1471-2164-9-78 (PMC2262897; doi:10.1186/1471-2164-9-78)
Supplement: Additional file 12 — Description of the 246 genes unique to the 50 nM regression analysis. Word document containing descriptions of the column headers found in Additional file 11. [file 1471-2164-9-78-S12.doc]

The 246 genes identified by the 50 nM regression analysis that were not identified by the 5 nM regression analysis. Sal-Site is an *Ambystoma* data repository that is publicly accessible on the World Wide Web (www.ambystoma.org).

Column A: Unique probe-set ID for probe-sets on the custom *Ambystoma* GeneChip

Column B: Sal-Site contig name

Column C: Sal-Site contig identifier

Column D: The best human BLASTX hit to a salamander contig query

Column E: E-value for the BLASTX search described for the previous column

Column F: RefSeq identifer for human hits associated with BLASTX searches

Column G: Name of the human hit associated with BLASTX searches

Column H: Entrez gene identifier of the best human hit associated with BLASTX searches

Column I: URL associated with the Entrez gene identifier mentioned for previous the column

Column J: The rank of a given probe-set in the 5 nM regression analysis based on the over-all *P*-value (1 corresponds to the smallest *P*-value)

Column K: The FDR adjusted significance threshold in the 5 nM regression analysis against which the overall *P*-values are assessed

Column L: *P*-value from the 5 nM regression analysis associated with the overall model fit to a given probe-set

Column M: *P*-value from the 5 nM regression analysis associated with the quadratic term in a model fit to a given probe-set

Column N: *P*-value form the 5 nM regression analysis associated with the linear term in a model fit to a given probe-set

Column O: The intercept from the 5 nM regression analysis of a model fit to a given probe-set

Column P: Coefficient from the 5 nM regression analysis for the linear term in a model fit to a given probe-set

Column Q: Coefficient from the 5 nM regression analysis for the quadratic term in a model fit to a given probe-set

Column R: The expression pattern observed for a given probe-set in the 5 nM regression analysis

Column S: The generalized direction of expression observed in the 5 nM regression analysis. zs = not significant (for sorting purposes)

Column T: The directional trend assigned to probe-sets that were not statistically significant in the 5 nM analysis

Column U: Logical statement describing whether the model fit to a given probe-set is statistically significant in the 5 nM regression analysis upon adjusting the FDR of α_0_ to 0.05 (yes = significant)

Column V: The rank of a given probe-set in the 50 nM regression analysis based on the over-all *P*-value (1 corresponds to the smallest *P*-value)

Column W: The FDR adjusted significance threshold in the 50 nM regression analysis against which the overall *P*-values are assessed

Column X: *P*-value from the 50 nM regression analysis associated with the overall model fit to a given probe-set

Column Y: *P*-value from the 50 nM regression analysis associated with the quadratic term in a model fit to a given probe-set

Column Z: *P*-value form the 50 nM regression analysis associated with the linear term in a model fit to a given probe-set

Column AA: The intercept from the 50 nM regression analysis of a model fit to a given probe-set

Column AB: Coefficient from the 50 nM regression analysis for the linear term in a model fit to a given probe-set

Column AC: Coefficient from the 50 nM regression analysis for the quadratic term in a model fit to a given probe-set

Column AD: The expression pattern observed for a given probe-set in the 50 nM regression analysis

Column AE: Generalized direction of expression observed in the 50 nM regression analysis

Column AF: Logical statement describing whether the model fit to a given probe-set is statistically significant in the 50 nM regression analysis upon adjusting the FDR of α_0_ to 0.05 (yes = significant)

Columns AG-AM: Back-transformed (raw scale) mean values for Day 0 controls and the non-control treatment by sampling time groups.

Columns AN-AS: Back-transformed (raw scale) fold change values for each non-control treatment by sampling time. The non-control is in the numerator and Day 0 is in the denominator. Values of 1 indicate that expression is identical to Day 0 values > 1 indicate up-regulation relative to Day 0 and values < 1 indicate down regulation relative to Day 0.

Column AT: Logical statement indicating whether a 2 fold change was observed relative to Day 0 controls in any non-control sampling time in the 5 nM dataset.

Column AU: Logical statement indicating whether a 2 fold change was observed relative to Day 0 controls in any non-control sampling time in the 50 nM dataset.

Column AV: Logical statement indicating whether the same generalized direction of expression was observed in the 5 and 50 nM T_4_ treatments.

Column AW: Logical statement indicating whether a given gene was classified as a late response gene in the 50 nM dataset.
