# Supplementary material for: Effect of thyroid hormone concentration on the transcriptional response underlying induced metamorphosis in the Mexican axolotl (Ambystoma)
Source: BMC Genomics. 2008 Feb 11;9:78. doi: 10.1186/1471-2164-9-78 (PMC2262897; doi:10.1186/1471-2164-9-78)
Supplement: Additional file 14 — Description of the 111 genes identified as differentially expressed in axolotl epidermis and Xenopus intestine. Word document containing descriptions of the column headers in Additional file 13. [file 1471-2164-9-78-S14.doc]

The 111 genes identified as differentially expressed in axolotl epidermis and *Xenopus* intestine. Sal-Site is an *Ambystoma* data repository that is publicly accessible on the World Wide Web (www.ambystoma.org).

Column A: Unique probe-set ID for probe-sets on the custom *Ambystoma* GeneChip

Column B: Sal-Site contig name

Column C: Sal-Site contig identifier

Column D: The best human BLASTX hit to a salamander contig query

Column E: E-value for the BLASTX search described for the previous column

Column F: RefSeq identifer for human hits associated with BLASTX searches

Column G: Name of the human hit associated with BLASTX searches

Column H: Entrez gene identifier of the best human hit associated with BLASTX searches

Column I: URL associated with the Entrez gene identifier mentioned for previous the column

Column J: Logical statement indicating whether a gene was identified as statistically significant among any of the contrasts performed in the concentration analysis (FALSE = significant in one or more contrasts)

Columns K-M: Logical statement indicating whether a gene was significant in a contrast performed at a particular sampling time (yes = significant)

Columns N-P: Fold change values observed (50/5) between the two T_4_ treatments at Days 2, 12, and 28 respectively.

Column Q: Logical statement indicating whether a given gene was statistically significant and differentially regulated by ≥ two-fold relative to Day 0 controls in the 5 nM regression analysis.

Column R: Regression pattern observed in the 5 nM analysis (Z/A = not identified by this analysis)

Columns S-U: Fold change values observed in the 5 nM treatment relative to Day 0 controls.

Column V: Logical statement indicating whether a given gene was statistically significant and differentially regulated by ≥ two-fold relative to Day 0 controls in the 50 nM regression analysis.

Column W: Regression pattern observed in the 50 nM analysis (Z/A = not identified by this analysis)

Column X: Generalized direction of differential expression observed in our experiment (z = only significant in the concentration analysis)

Column Y-AA: Fold change values observed in the 50 nM treatment relative to Day 0 controls.

Column AB: GenBank identifier associated with a *Xenopus* sequence (gene)

Column AC: Name of a *Xenopus* gene

Column AD: Name of human GI identifier associated with best BLASTX hit to a *Xenopus* query

Column AE: RefSeq identifier associated with the best human BLASTX hit to a *Xenopus* query

Column AF: E-value associated with a human BLASTX hit to a *Xenopus* query

Column AG: Name of the best human BLASTX hit to a *Xenopus* query

Column AH: Entrez Gene identifier associated with the best human BLASTX hit to a *Xenopus* query

Column AI-AK: Fold changes observed in the intestine of metamorphosing *Xenopus* by Buchholz et al. (2007) following 1, 3, and 6 days of treatment with T_3_.

Column AL: Generalized direction of differential expression (as determined by the maximum fold change value) observed in *Xenopus*

Column AM: Logical statement indicating whether a given gene was listed by Buchholz et al. (2007) as up-regulated by ≥ 1.5 fold in brain, tail, limb, and intestine from metamorphosing *Xenopus*

Column AN: Logical statement indicating whether a gene exhibited the same generalized direction of differential expression in axolotl epidermis and *Xenopus* intestine. TRUE = same direction in both species
